# Near-infrared photoimmunotherapy in cancer treatment: a bibliometric and visual analysis

**DOI:** 10.3389/fphar.2024.1485242

**Published:** 2024-10-21

**Authors:** Jinglin Tian, Chunbao Chen, Xue Du, Miao Wang

**Affiliations:** ^1^ Department of Pharmacy, Suzhou Kowloon Hospital, Shanghai Jiaotong University School of Medcine, Suzhou, Jiangsu, China; ^2^ Department of Neurosurgery, The 3RD Affiliated Hospital of Chengdu Medical College, Pidu District People's Hospital, Chengdu, Sichuan, China; ^3^ Department of Clinical Medicine, North Sichuan Medical College, Nanchong, Sichuan, China; ^4^ Department of Oncology, Siyang Hospital, Suqian, Jiangsu, China

**Keywords:** near-infrared photo-immunotherapy, NIR-PIT, cancer, photosensitizer, bibliometrics, visual analysis

## Abstract

**Background:**

Near-infrared photoimmunotherapy (NIR-PIT) is an emerging cancer treatment technology that combines the advantages of optical technology and immunotherapy to provide a highly effective, precise, and low side-effect treatment approach. The aim of this study is to visualize the scientific results and research trends of NIR-PIT based on bibliometric analysis methods.

**Methods:**

The Web of Science Core Collection (WoSCC) database was searched in August 2024 for relevant publications in the field of NIR-PIT. Data were analyzed using mainly CiteSpace and R software for bibliometric and visual analysis of the country/region, authors, journals, references and keywords of the publications in the field.

**Results:**

A total of 245 publications were retrieved, including articles (n = 173, 70.61%) and reviews (n = 72, 29.39%). The annual and cumulative number of publications increased every year. The highest number of publications was from the United States (149, 60.82%), followed by Japan (70, 28.57%) and China (33, 13.47%). The research institution with the highest number of publications was National Institutes of Health (NIH)-USA (114, 46.53%). Kobayashi H (109) was involved in the highest number of publications, Mitsunaga M (211) was the most frequently cited in total. CANCERS (17) was the most frequently published journal, and NAT MED (220) was the most frequently co-cited journal. The top 10 keywords include near-infrared photoimmunotherapy (166), photodynamic therapy (61), monoclonal antibody (58), *in vivo* (50), cancer (46), expression (31), breast cancer (27), enhanced permeability (24), antibody (23), growth factor receptor (16). Cluster analysis based on the co-occurrence of keywords resulted in 13 clusters, which identified the current research hotspots and future trends of NIR-PIT in cancer treatment.

**Conclusion:**

This study systematically investigated the research hotspots and development trends of NIR-PIT in cancer treatment through bibliometric and visual analysis. As an emerging strategy, the research on the application of NIR-PIT in cancer treatment has significantly increased in recent years, mainly focusing on the targeting, immune activation mechanism, and treatment efficacy in solid tumors has received extensive attention. Future studies may focus on improving the efficacy and safety of NIR-PIT in cancer treatment, as well as developing novel photosensitizers and combination therapeutic regimens, and exploring the efficacy of its application in a wide range of solid tumors, which will provide an important reference and guidance for the application of NIR-PIT in clinical translation.

## 1 Introduction

The International Agency for Research on Cancer (IARC) latest publication, “Global cancer burden growing, amidst mounting need for services,” once again emphasizes that the current growing global cancer burden deserves worldwide attention (Anonymous, 2024). The latest global cancer statistics reported in 2024 show a serious and growing global cancer burden. In 2022, there will be approximately 20 million new cancer cases and nearly 10 million cancer-related deaths worldwide. Lung cancer is the most commonly diagnosed type, accounting for 12.4% of all new cases, followed closely by breast (11.6%) and colorectal (9.6%) cancers. Lung cancer also leads the mortality rate, accounting for 18.7% of all cancer deaths, with colorectal and liver cancers being the second most lethal cancers (The American Cancer Society, 2024). With the incidence of cancer continuing to rise globally and cancer treatment as a focused field of medical research, the field of cancer treatment has undergone significant changes. Traditional treatments such as surgery, chemotherapy and radiotherapy suffer from a balance between therapeutic efficacy and side effects, and have limited effectiveness for certain cancer types. Therefore, the focus of research in recent years has gradually shifted to targeted therapy, immunotherapy, and other emerging therapies (Mun et al., 2018; Kaur et al., 2023). Near-Infrared Photoimmunotherapy (NIR-PIT) is a novel cancer treatment strategy that combines localized target-cell killing through a unique photo-induced ligand release reaction with the systemic immune activation characteristic of immunotherapy, demonstrating promising treatment efficacy and low side effects.

In 2011, Mitsunaga et al. (2011) first reported NIR-PIT, a novel molecularly targeted cancer treatment. Photoimmunotherapy (PIT) uses a target-specific photosensitizer based on IRDye700DX (IR700), conjugated to a monoclonal antibody (mAb) targeting the epidermal growth factor receptor (EGFR). Irradiation of mAb-IR700-bound target cells with NIR light immediately induces cell death. The distinct features of NIR-PIT are the specific targeting of antibodies to antigens on the surface of cancer cells as the most critical advantage to achieve high tumor specificity, as well as the laser-activated biophysical mechanism, which results in cell necrosis mainly through physical effector disruption of cell membranes (Kobayashi and Choyke, 2019). The characteristics of NIR, with a wavelength of 650–900 nm has a strong tissue penetration ability, and ability to reach deep into the tumour, thus achieving effective treatment of deep tumours (Sakudo, 2016). PIT combines the specific targeting ability of monoclonal antibodies and photosensitizing dyes, after local irradiation with NIR light, the photosensitizing dyes are activated, leading to rapid membrane damage and cell death of antibody-conjugated cancer cells, as well as the release of tumor antigens, which further activate the immune system and amplify the therapy effect of NIR-PIT. With the development and optimization of photosensitizers and specific targeting antibodies, NIR-PIT shows a promising clinical application.

The current clinical application of NIR-PIT is focused on solid tumors that are difficult to surgically resect and tumors that are resistant to conventional treatments. In late 2017, the first human Phase I/II clinical trial of NIR-PIT using cetuximab-IR700 (RM-1929) targeting the EGFR for the treatment of patients with inoperable head and neck squamous cell carcinoma (HNSCC) was successfully completed (https://clinicaltrials.gov/ct2/show/NCT02422979). NIR-PIT has shown significant results in the treatment of head and neck tumours, and this study also supports the potential of NIR-PIT in the treatment of solid tumours. A global Phase III clinical trial of NIR-PIT in patients with recurrent head and neck squamous cell carcinoma (rHNSCC) is currently underway (https://clinicaltrials.gov/ct2/show/NCT03769506). As of September 2020, conditional clinical approval for NIR-PIT for the treatment of rHNSCC has been approved in Japan (Maruoka et al., 2021). Yamaguchi et al. (2021) demonstrated that NIR-PIT using HER2 Affibody-IR700Dye conjugate and the trastuzumab-IR700Dye conjugate enhanced cytotoxic effects on HER2 positive breast cancer cells. This approach has the potential to improve the efficiency of current NIR-PIT, especially for heterogeneous HER2-positive cancers. Takao et al. (2023) showed that cetuximab IR700 NIR-PIT has a significant anti-tumor effect on EGFR-positive hepatocellular carcinoma (HCC), providing a potential strategy for HCC treatment. NIR-PIT can improve the overall therapeutic outcome of patients by increasing the immunogenicity of tumours and promoting anti-tumour immune responses (Kobayashi et al., 2021). The main advantage of NIR-PIT consists of its high targeting, which precisely locates the cancer cells through antibody-photoabsorber conjugate (APC), allowing the treatment to have minimal damage to the normal tissues and dramatically reducing the side-effects of the traditional treatments (Mohiuddin et al., 2023). NIR-PIT exhibits potential therapeutic efficacy in many types of cancers, such as glioblastoma (GBM) (Burley et al., 2018), pancreatic cancer (Maawy et al., 2015), lung cancer (Takahashi et al., 2021), and gastric cancer (Nishimura et al., 2020), which will hopefully further expand its clinical applications.

Bibliometrics is a quantitative analysis methodology for studying scientific literature, which mainly analysis the publication and citation patterns of literature by mathematical and statistical means in order to assess the output and impact of research activities as well as the patterns of knowledge dissemination (Rousseau, 2014). Bibliometrics is used to analysis the number, publication trends and field distribution of academic literature to understand the development of different disciplines or research themes. It can reveal research hotspots, identify scientific frontiers, and predict future trends. In this study, bibliometric analysis is used to understand the development of this emerging technology in the field of NIR-PIT, which provides a macroscopic view of the research development in this field, thus providing a more targeted research strategy to promote scientific and technological progress.

## 2 Materials and methods

### 2.1 Data source

Web of Science Core Collection (WoSCC) database contains a wide range of important academic journals in various disciplines around the world. As a key tool for researchers to access global academic information, it enables researchers to track academic development, discover frontier research, and conduct bibliometric analysis.

**FIGURE 1:**
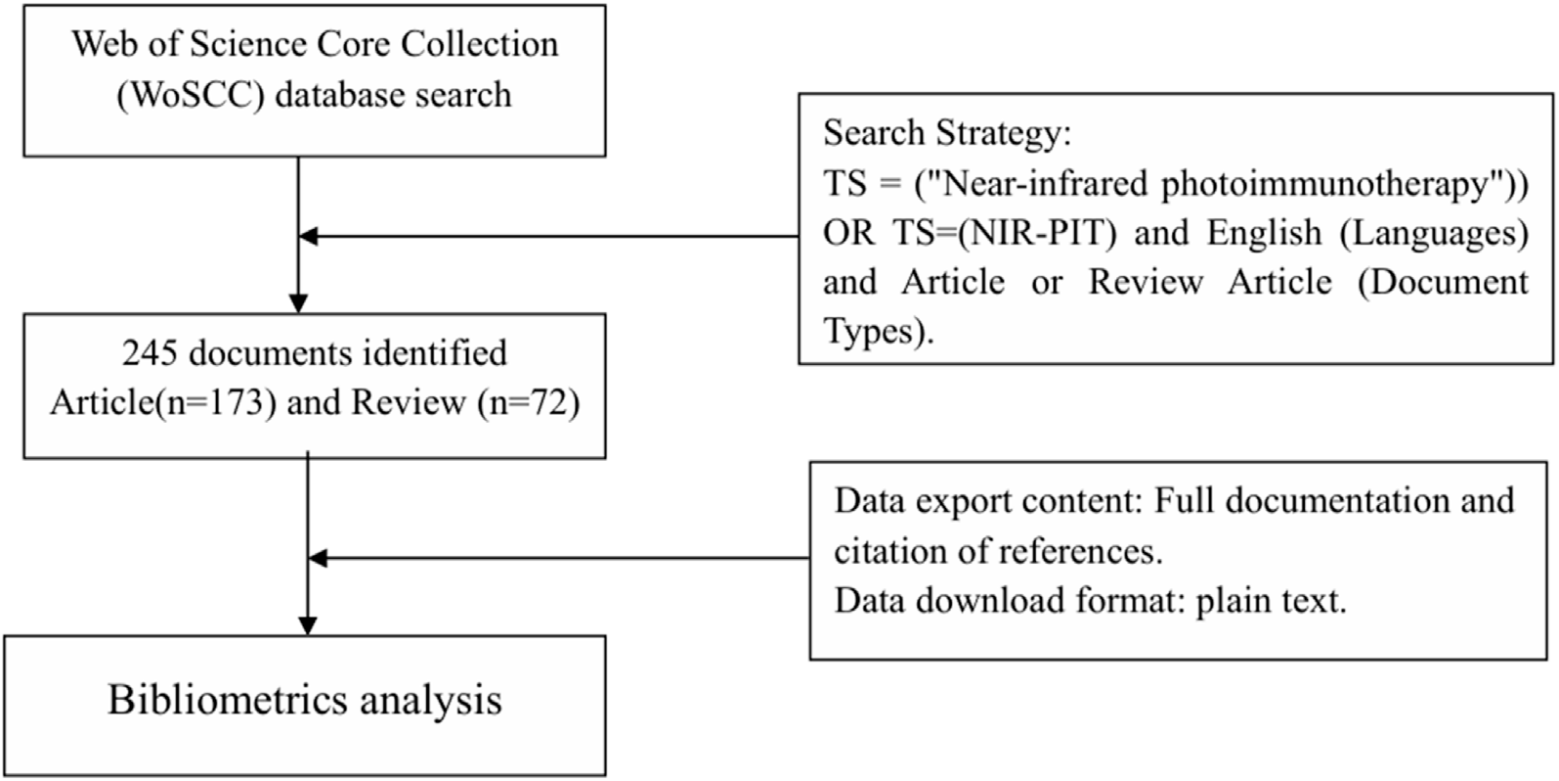
Shows a flow chart of document retrieval.

In August 2024, we searched the WoSCC database for documents related to the field of NIR-PIT research, with the timespan: 2011–2024. The search strategy: TS = (Near-infrared photoimmunotherapy) OR TS = (NIR-PIT) and English (Languages) and Article or Review Article (Document Types). The language: English. Document type: Article or Review article. Data export content: complete record and cited references. Data download format “Plain text.” Figure 1 shows the flow chart of document search.

### 2.2 Visual analysis

This study was conducted to analyze the bibliometrics and visual of documents related to the research field of NIR-PIT mainly through CiteSpace (version 6.2. R3) and R (version 4.3.3) software. Microsoft Excel 2019 was used for data management and trend analysis of publications. CiteSpace software is a bibliometric software developed by Prof. Chen, and its core function is to demonstrate the historical development and structural changes of a discipline through visual excavation and analysis of scientific and technological texts in a “knowledge domain” (Chen, 2006). Centrality is a measure of the importance or influence of a document or node in the whole network, including betweenness centrality, degree centrality and closeness centrality. Betweenness centrality is a measure of the importance of a node in a network and indicates the frequency of the shortest path through the node in a network. In a network, the greater the intermediary centrality of a node, the greater the role it plays in the communication between other nodes, acting as a communication bridge (Brandes, 2001). Generally centrality ≥0.1 is considered as more important nodes, in CiteSpace network mapping, this index is used to discover and measure the importance of the document and the high centrality is highlighted with a purple circle. Burst detection in CiteSpace is used to detect sudden changes in references and keywords.

Using the “bibliometrix” package in the R software, the source journals of the publications were performed a visual analysis to show intuitively the journals with the largest number of related publications in the field of NIR-PIT research, indicating that the journal is very active in publishing in this field.

## 3 Result

### 3.1 Publishing trend

**FIGURE 2:**
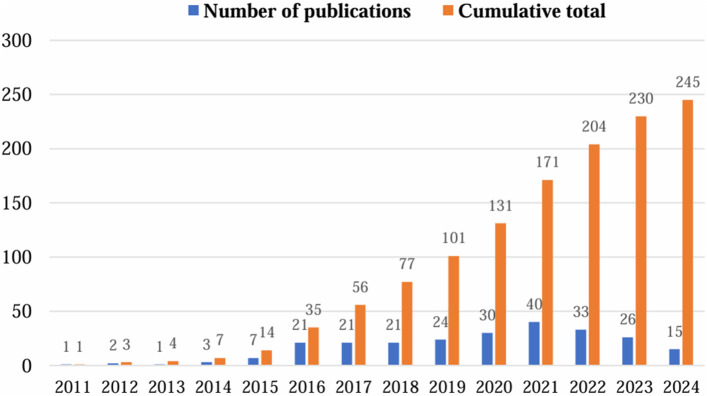
Trends in the number of relevant publications published.

A total of 245 documents related to the field of NIR-PIT were retrieved in WoSCC according to the set search strategy, including articles (173, 70.61%) and review article (72, 29.39%). Supplementary Table S1 shows the annual and cumulative number of publications. Figure 2 shows the trend chart of the number of publications. The change of the annual number of publications reflects the dynamic change of research hotspots, while the cumulative number of publications shows the long-term development trend of the field. Research on the field of NIR-PIT was first reported in 2011, and at the beginning of the research period, the number of annual publications was relatively low, probably due to the fact that the field of research was just emerging or had a low level of attention. Over time, the number of annual publications gradually increased until 2016, when the number of annual publications exceeded 20 and rose steadily, indicating that the research activities in this field are becoming active and the academic attention has increased significantly. Overall, the cumulative number of publications shows a trend of steady growth, indicating that the research field is gradually maturing and gaining signs of widespread attention.

### 3.2 Country/region and institution

**FIGURE 3:**
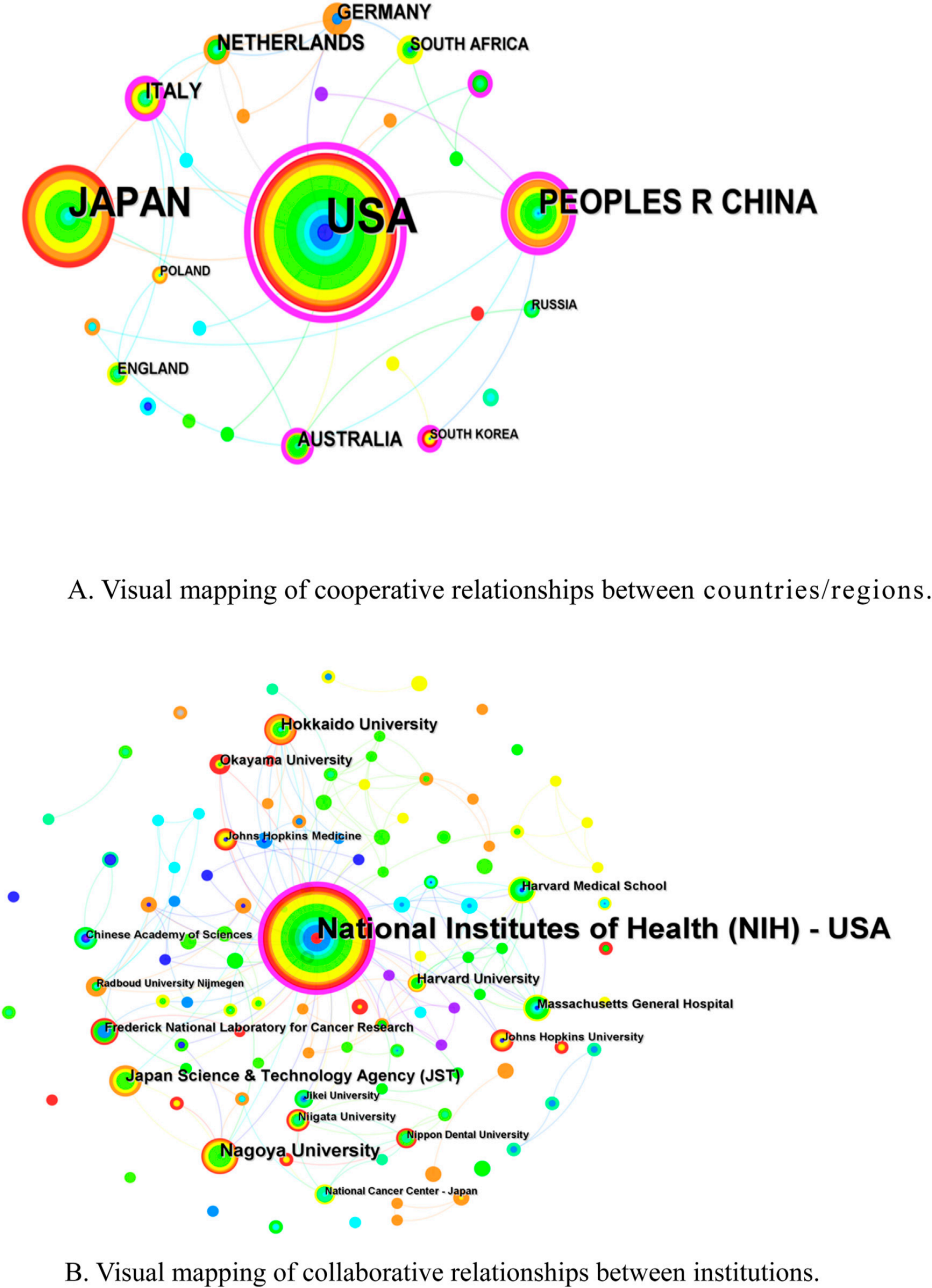
**(A)** Visual mapping of cooperative relationships between countries/regions. **(B)** Visual mapping of collaborative relationships between institutions.

A total of 26 countries/regions with 233 institutions, involved in the publication of relevant literature in the field of NIR-PIT, were obtained after screening. The highest number of publications was from the United States (149, 60.82%), followed by Japan (70, 28.57%) and China (33, 13.47%) (Table 1). The United States has the highest centrality (0.98), followed by China (0.43), Italy (0.25) and Australia (0.20). Figure 3A presents a visual network mapping of the collaborative relationships across countries/regions. The nodes in the network mapping represent different countries/regions, and the lines represent the co-operation between these countries/regions. The size of the node reflects the number of publications in the country/region, the line thickness reflects the strength of cooperation, and the purple circle outside the node represents the high centrality, the greater centrality of a node, the greater role that it plays in the communication between other nodes. Among them, the United States works most closely with other countries, including Japan, Germany, Italy, China, and South Korea.

**TABLE 1:** Top 10 country/region for relevant publications.

Rank	Countries	Count	Centrality	% of 245
1	UNITED STATES	149	0.98	60.82
2	JAPAN	70	0.01	28.57
3	PEOPLES R CHINA	33	0.43	13.47
4	ITALY	8	0.25	3.27
5	NETHERLANDS	8	0.08	3.27
6	GERMANY	7	0.07	2.86
7	AUSTRALIA	7	0.2	2.86
8	SOUTH AFRICA	5	0.03	2.04
9	ENGLAND	4	0	1.63
10	SOUTH KOREA	3	0.13	1.22

The research institution with the highest number of publications was National Institutes of Health (NIH)-USA (114, 46.53%) and had the highest centrality (0.22), followed by Nagoya University (15) (Supplementary Table S2). Figure 3B shows the collaborative network between institutions. The nodes represent institutions and the lines represent the collaborative relationships between these institutions. (NIH)-USA collaborate most closely with each other and occupy a central position in the collaboration network, indicating that they play a leading role in collaboration in this field.

### 3.3 Author and co-cited author

**FIGURE 4:**
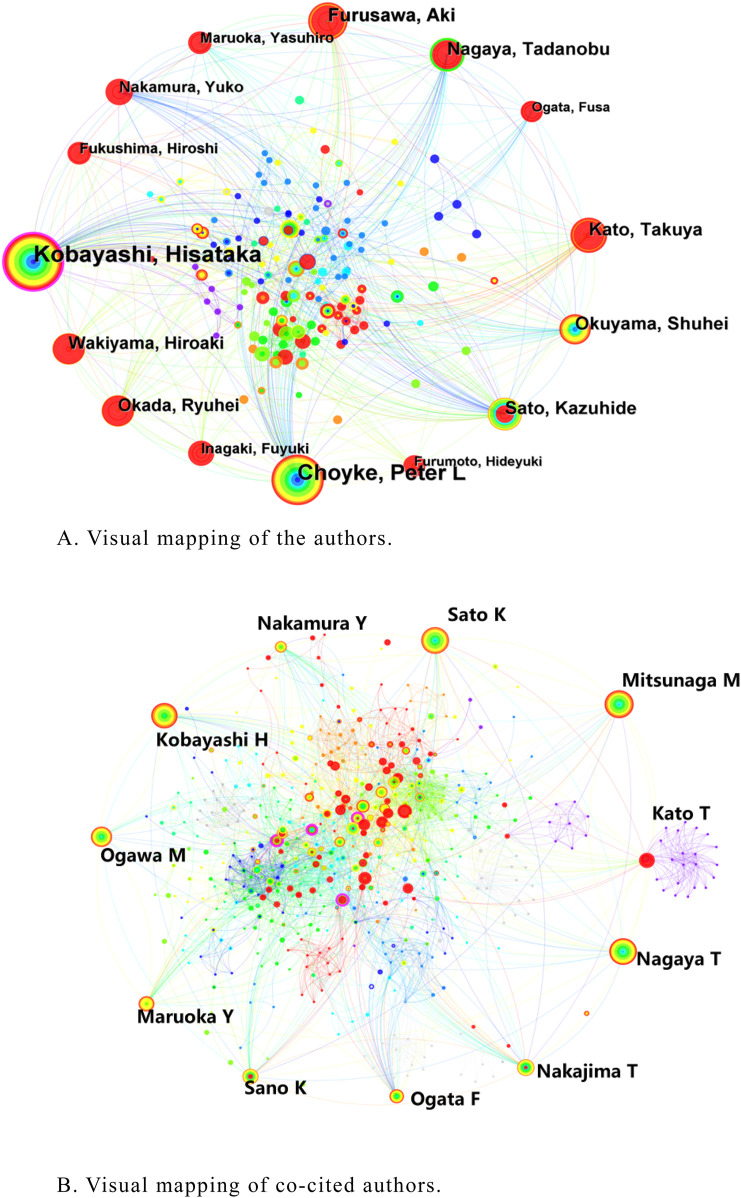
**(A)** Visual mapping of the authors. **(B)** Visual mapping of co-cited authors.

A total of 372 researchers were contributing to the publication of the relevant documents. The most published author was Kobayashi H (109), followed by Choyke PL (90), and Furusawa A (41). Kobayashi H had the highest centrality (0.16) among the top 10 authors (Table 2). Figure 4A shows the author collaboration network visual mapping. The nodes represent different authors and the lines represent the collaborations between these authors. The size of the nodes reflects the number of publications by the authors, and the thickness of the lines indicates the frequency of collaboration. Kobayashi H and Choyke PL collaborate most closely with other authors, and they play an important role in promoting research progress and innovation.

**TABLE 2:** The top 10 authors and co-cited authors for relevant publications.

Rank	Author	Count	Centrality	Co-cited author	Count	Centrality
1	Kobayashi H	109	0.16	Mitsunaga M	211	0.05
2	Choyke PL	90	0.07	Sato K	185	0.01
3	Furusawa A	41	0	Nagaya T	144	0.01
4	Sato K	38	0.05	Kobayashi H	132	0.02
5	Nagaya T	37	0	Ogawa M	99	0.01
6	Kato T	36	0.01	Nakajima T	77	0.03
7	Okuyama S	34	0.01	Sano K	76	0.02
8	Okada R	31	0	Maruoka Y	60	0.03
9	Wakiyama H	29	0	KatoT	48	0.01
10	Inagaki F	19	0	Nakamura Y	46	0.06

Author co-citation analysis (ACA) refers to the fact that two or more authors are cited in one or more articles at the same time, and these two or more authors constitute a co-citation relationship (White and McCain, 1998). The most cited author was Mitsunaga M (211), followed by Sato K (185), Nagaya T (144) (Table 2). Figure 4B shows the network of co-cited authors, where the size of the nodes represents the authors’ citation frequency and the lines between the nodes represent the strength of the co-citation relationship. The analysis of author co-citations reveals important academic figures and the influence of their research within the NIR-PIT research field.

### 3.4 Journal and co-cited journal

**FIGURE 5:**
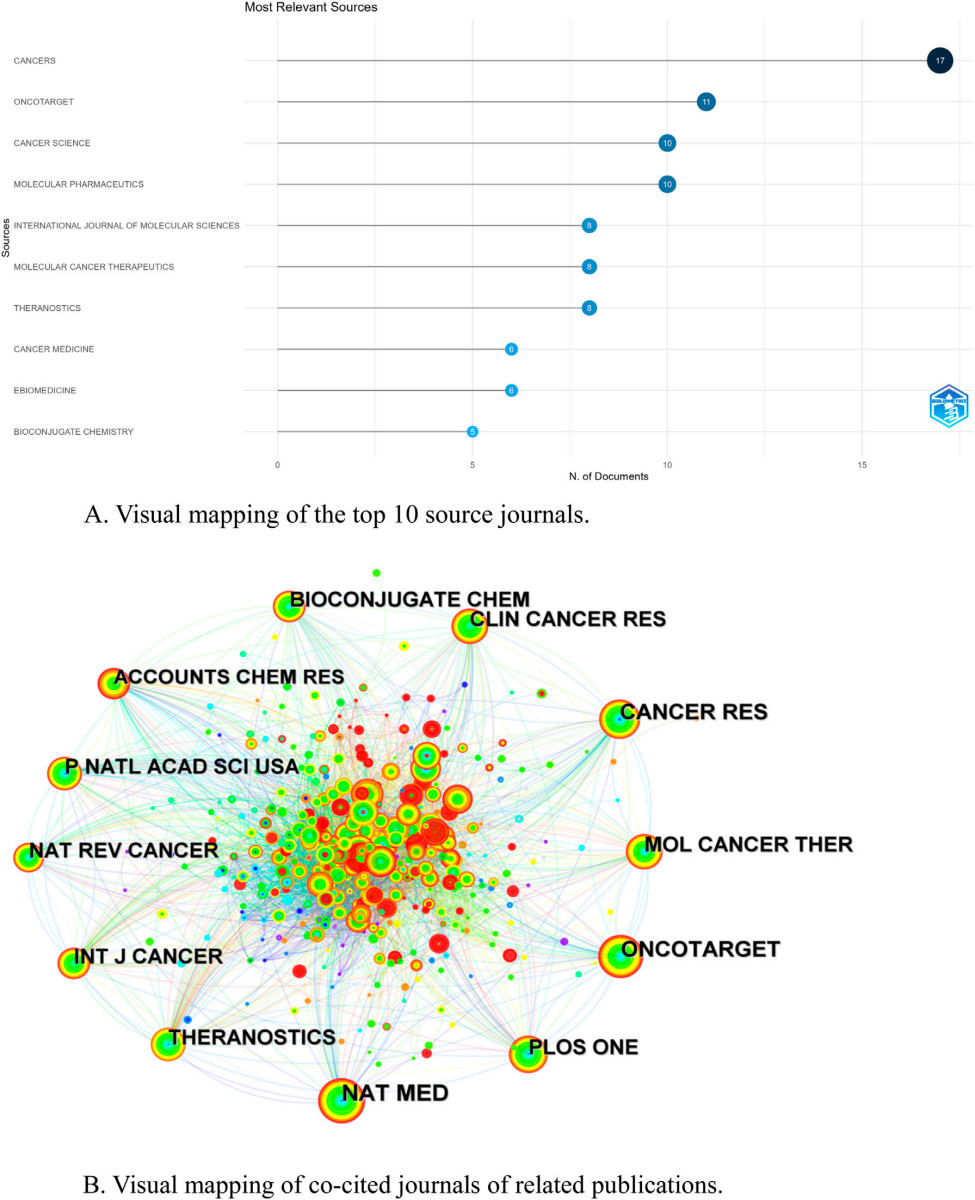
**(A)** Visual mapping of the top 10 source journals. **(B)** Visual mapping of co-cited journals of related publications.

Journal visual analysis can reveal the core journals in a certain field, which usually have high academic impact and publication frequency in the field. The published journals were analyzed visually using the “bibliometrix” package of the R software. A total of 117 academic journals published documents related to the field of NIR-PIT, and the journal with the highest number of publications was CANCERS (17), followed by ONCOTARGET (11), CANCER SCIENCE (10), and MOLECULARE PHARMACEUTICS (10) (Table 3). Figure 5A shows the visual mapping of academic journals ranked top 10 in the number of publications, among which the journal with the highest impact factor (IF) is THERANOSTICS (IF 12.4), followed by EBIOMEDICINE (IF 9.7) and MOLECULAR CANCER THERAPEUTICS (IF 5.3). The most frequently cited journal among the 405 co-cited journals was NAT MED (220), followed by ONCOTARGET (179) and CANCER RES (165) (Table 3). Among the top 10 co-cited journals, NAT MED had the highest impact factor (IF 58.7), followed by CANCER RES (IF 12.5) and THERANOSTICS (IF 12.4), suggesting that these journals have a high influence in this research field. Journal co-citation is reflected by the relevance of various journals and disciplines, Figure 5B shows the network of co-cited journals, the nodes represent the journals and the size of the nodes represents the number of citations. The line represents the strength of the co-citation relationship, the thicker the line, the stronger the co-citation relationship between two journals.

**TABLE 3:** Top 10 Sources journal and co-cited journals.

Rank	Count	Sources journal	IF 2024	JCR	Co-cited journals	Count	IF 2024	JCR
1	17	CANCERS	4.5	Q1	NAT MED	220	58.7	Q1
2	11	ONCOTARGET	0	0	ONCOTARGET	179	0	0
3	10	CANCER SCIENCE	4.5	Q1	CANCER RES	165	12.5	Q1
4	10	MOLECULAR PHARMACEUTICS	4.5	Q1	PLOS ONE	147	2.9	Q3
5	8	INTERNATIONAL JOURNAL OF MOLECULAR SCIENCES	4.9	Q1	MOL CANCER THER	139	5.3	Q1
6	8	MOLECULAR CANCER THERAPEUTICS	5.3	Q1	CLIN CANCER RES	139	10	Q1
7	8	THERANOSTICS	12.4	Q1	THERANOSTICS	133	12.4	Q1
8	6	CANCER MEDICINE	2.9	Q2	BIOCONJUGATE CHEM	132	4	Q1
9	6	EBIOMEDICINE	9.7	Q1	INT J CANCER	128	5.7	Q1
10	5	BIOCONJUGATE CHEMISTRY	4	Q1	NAT REV CANCER	125	72.5	Q1

Dual map overlay of journals refers to the overlapping presentation of two journal network maps, an analytical tool used to visualize inter-journal relationships and domain evolution, thus revealing trends and dynamic relationships between journals (Chen and Loet, 2014). The left side is the sizing citation graph and the right side is the corresponding cited graph. The curves are citation connectors, showing the full context of the citations. In Supplementary Figure S1 the colored paths indicate citation relationships, the yellow paths indicate that documents published in (Molecular/Biology/Immunology) journals are frequently cited by (Chemistry/Materials/Physics) journals. The green path indicates that documents published in (molecular/medical/clinical) journals are often cited in (Environmental/Toxicology/Nutrition) journals. Purple paths indicate that documents published in (Chemistry/Materials/Physics) journals are frequently cited by (Health/Nursing/Medicine) journals. The analysis of the journal overlay map reveals the cross-citation relationship between different journals and understands the intersection and integration between the fields.

### 3.5 Co-cited reference and reference burst

**FIGURE 6:**
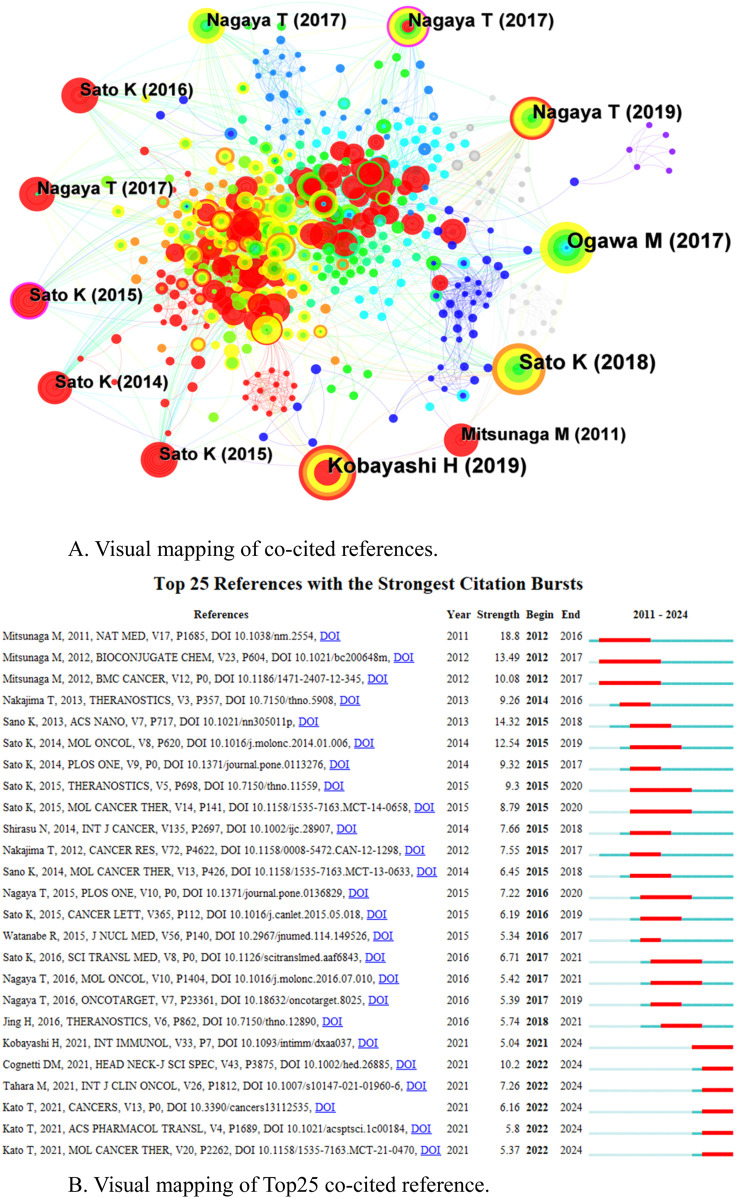
**(A)** Visual mapping of co-cited references. **(B)** Visual mapping of Top25 co-cited reference.

Among the 543 co-cited references, Table 4 shows the top 10 references in terms of co-citation frequency. One of the most frequently cited publications is the first comprehensive review article on newly developed photochemistry-based cancer therapy near-infrared (NIR) photoimmunotherapy (PIT) by Kobayashi H in 2019 (Mitsunaga et al., 2011). A comprehensive overview of the mechanisms, clinical research results and future progress of NIR-PIT for cancer treatment. Figure 6A shows the network of co-cited references, with the nodes represent a co-cited reference, the size of the node represents the citation frequency of the document, the thickness of the line represents the frequency of co-citation, the purple ring represents having a high centrality, and the red circle represents burst. Citation burst analysis (CBA) is used to identify documents that suddenly receive a large number of citations within a specific time period, which usually marks a key breakthrough or emerging hotspot in a research field (Kleinberg, 2002). Based on the strongest citation bursts, Figure 6B shows that the first citation burst started in 2011, Mitsunaga et al. (2011) and Kobayashi and Choyke (2019) first reported “Cancer cell-selective *in vivo* near-infrared photoimmunotherapy targeting specific membrane molecules,” which represents a key research achievement and breakthrough in the field of NIR-PIT. The citation burst strengths of the top 25 references range from 5.04–18.8, with five of them bursting from 2021 to the present.

**TABLE 4:** Top 10 co-cited references of related publications.

Rank	Year	Count	Centrality	Cited reference
1	2019	90	0.01	Kobayashi H, 2019. Near-Infrared Photoimmunotherapy of Cancer
2	2018	82	0.04	Sato K, 2018. Photoinduced Ligand Release from a Silicon Phthalocyanine Dye Conjugated with Monoclonal Antibodies: A Mechanism of Cancer Cell Cytotoxicity after Near-Infrared Photoimmunotherapy
3	2017	79	0.05	Ogawa M, 2017. Immunogenic cancer cell death selectively induced by near infrared photoimmunotherapy initiates host tumor immunity
4	2019	56	0.08	Nagaya T, 2019. Host Immunity Following Near-Infrared Photoimmunotherapy Is Enhanced with PD-1 Checkpoint Blockade to Eradicate Established Antigenic Tumors
5	2017	45	0.13	Nagaya T, 2017. Near infrared photoimmunotherapy with avelumab, an anti-programmed death-ligand 1 (PD-L1) antibody
6	2017	40	0.03	Nagaya T, 2017. Near-Infrared Photoimmunotherapy Targeting Prostate Cancer with Prostate-Specific Membrane Antigen (PSMA) Antibody
7	2017	38	0.05	Nagaya T, 2017. Syngeneic Mouse Models of Oral Cancer Are Effectively Targeted by Anti-CD44-Based NIR-PIT
8	2015	37	0.02	Sato K, 2015. Near infrared photoimmunotherapy in the treatment of pleural disseminated NSCLC: preclinical experience
9	2016	37	0.03	Sato K, 2016. Spatially selective depletion of tumor-associated regulatory T cells with near-infrared photoimmunotherapy
10	2015	35	0.1	Sato K, 2015. Near infrared photoimmunotherapy in the treatment of disseminated peritoneal ovarian cancer

### 3.6 Keyword co-occurrence and clustering

**FIGURE 7:**
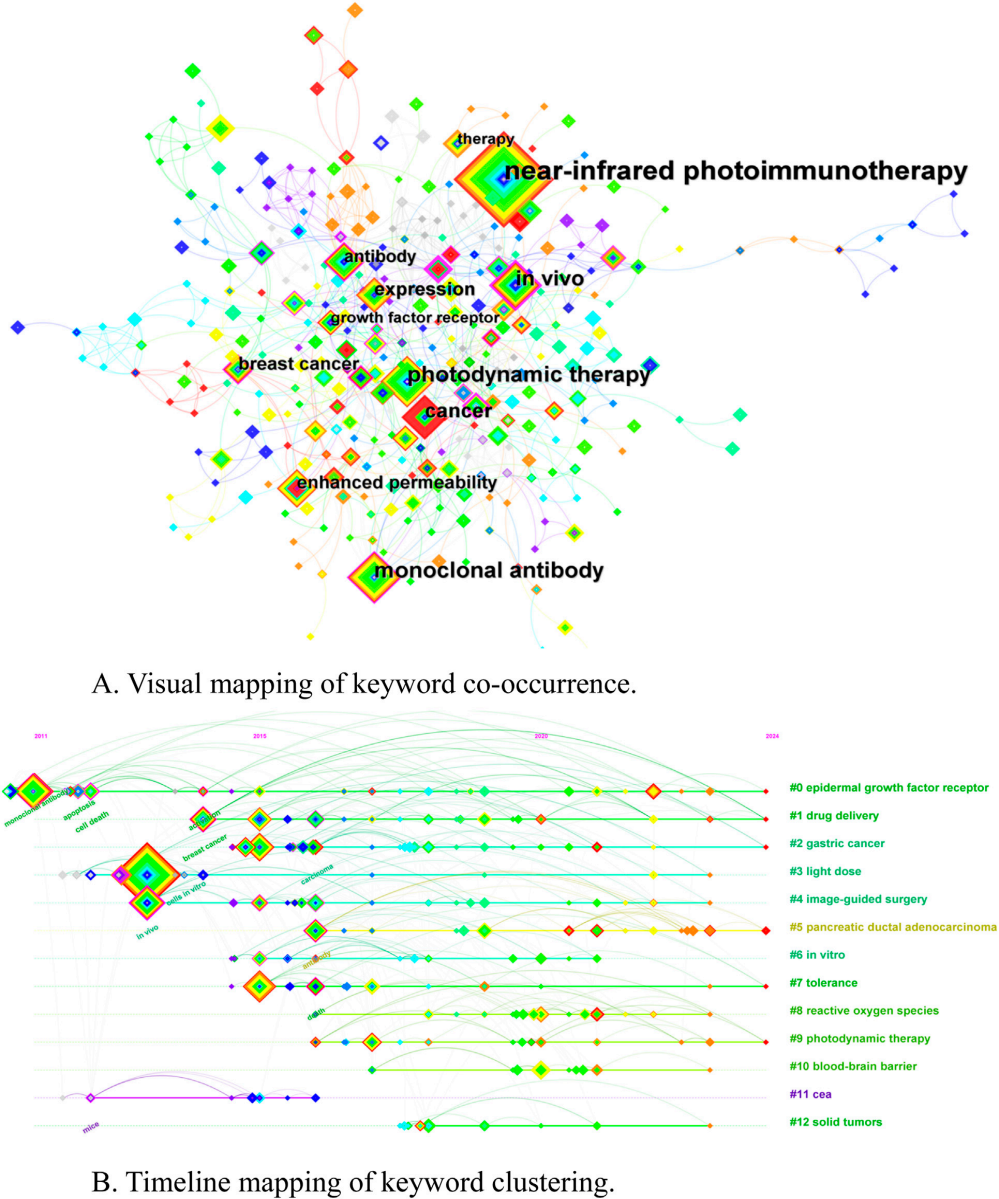
**(A)** Visual mapping of keyword co-occurrence. **(B)** Timeline mapping of keyword clustering.

Co-occurrence analysis is based on the analysis of common occurrences of words or noun phrases in a collection of documents to construct a network of associations between words and to reveal the relationships between different topics and concepts (Hu and Zhang, 2017). The keywords of the documents are the words or phrases used to describe the theme, research content or field of the documents, which can reflect the core theme and research range of the documents. Keyword co-occurrence analysis can help identify hot issues in the field, and frequently co-occurring keywords usually indicate current research hotspots (Supplementary Table S3). Shows the top 10 keywords in the documents related to the field of NIR-PIT, including near-infrared photoimmunotherapy (166), photodynamic therapy (61), monoclonal antibody (58), *in vivo* (50), cancer (46), expression (31), breast cancer (27), enhanced permeability (24), antibody (23), growth factor receptor (16). Figure 7A shows the visual mapping of keyword co-occurrence, the nodes represent the keywords, and the color and size of the nodes indicate the importance and frequency of the keywords. Keyword clustering analysis was performed on the basis of keyword co-occurrence analysis, and the research hotspots within the field can be refined by analyzing the high-frequency co-occurring keyword clusters. Supplementary Figure S2 shows a virtual map of the keyword cluster analysis, with 13 clusters formed by the main NIR-PIT research field: #0 epidermal growth factor receptor, #1 drug delivery, #2 gastric cancer, #3 light dose, #4 image-guided surgery, #5 pancreatic ductal adenocarcinoma, #6 *in vitro*, #7 tolerance, #8 reactive oxygen species, #9 photodynamic therapy, #10 blood-brain barrier, #11 cea, #12 solid tumors, with Cluster ID being the number after clustering, which is shown in the figure as #0, #1, etc. The larger the size of the cluster and the larger the number of members included in the cluster, the smaller the number. Figure 7B shows the timeline mapping of keyword clustering, enabling an in-depth exploration of keyword clustering in the time dimension, demonstrating the evolution of keywords and changes in clustering over time.

Further keyword burst analysis is carried out, to clearly show the sudden changes and hot trends of keywords in the research field, allowing us to understand the current research dynamics, identify new research hotspots, and provide valuable references for the future research direction. Supplementary Figure S3 shows the keyword burst mapping, revealing the burst intensities of the top 24 keywords in NIR-PIT related fields. The first burst started in 2011, with burst strengths ranging from 1.93–3.71, with membrane (3.71) having the biggest burst strength. The lengths of the red lines represent the duration of the bursts. Ovarian cancer and apoptosis are the keywords with the longest duration of the bursts, while membrane, cancer and therapy are the keywords with the longest duration of the bursts from 2022 to the present, indicating that this is the current hotspot of the research, and that future studies can further explore the potential applications and development trends in this field.

## 4 Discussion

### 4.1 General information

To some extent, the number of publications is an important indicator for assessing academic research activities and trends. This study reveals the research dynamics and long-term trends in the field of NIR-PIT research by analyzing the annual and cumulative number of publications in the field. In 2011, the first article in the field of NIR-PIT research was published, Mitsunaga et al. (2011) and Kobayashi and Choyke (2019) first reported that NIR-PIT is a newly developed cancer therapy that uses an APC activated by irradiation with NIR light. Target-selective PIT is able to treat cancer based on mAb binding to the cell membrane. The annual and cumulative number of publications related to the field of NIR-PIT show a steady growth in the trend of change over the analyzed timeframe. The sustained growth in the number of publications indicates that the field has gradually matured, with more and more researchers investing in the field, which may lead to more interdisciplinary collaborations and research opportunities as the academic influence continues to expand.

In the country/region network analysis, United States, Japan and China occupy a central position in the collaborative network, suggesting that these countries/regions have significant academic influence in the field. United States has the highest centrality and is involved in the greatest number of international collaborations, and with closer cooperation between countries/regions may lead to more resource sharing and knowledge transfer, thus contributing to the overall advancement of the field. The institutions with the largest number of publications are also from the National Institutes of Health (NIH) in the United States, suggesting that they have significant academic influence in the field and may dominate key research themes and findings. In addition, enhanced support for collaboration between core institutions, particularly in the priority research fields, is recommended to foster deeper collaboration and innovation.

The collaborative network between authors and the close cooperation between core authors may have promoted key research advances in the field. Kobayashi H, as the researcher with the highest number of publications involved, has reported several findings in the field of NIR-PIT therapy for cancer (Kobayashi and Choyke, 2019; Sato et al., 2015), demonstrating positive therapy perspectives in a wide range of cancer types. In addition, the immune mechanism of NIR-PIT for cancer treatment is introduced in detail, photochemical reactions are proposed as a major mechanism of cell death induced by NIR-PIT, it can dramatically alter the physical properties of the reactive molecules (Sato et al., 2018). During treatment, antigens and cellular debris released by cancer cell destruction are recognized by the host immune system, which activates immune cells. This immune activation not only helps to destroy residual cancer cells, but may also generate long-term immune memory, thereby reducing the risk of tumor recurrence (Nagaya et al., 2019). These findings provide the theoretical basis and methodological support for subsequent studies, and make remarkable academic contributions to the development of the field of NIR-PIT. Mitsunaga M, as the researcher with the highest co-citation frequency, demonstrates that his findings have been widely recognized and cited in the field, contributing greatly to the use of NIR-PIT in the treatment of cancer progression, confirmed that PIT induces massive cell death in targeted tumour cells immediately after exposure of NIR light (Mitsunaga et al., 2012).

Journal analysis provides insights into academic fields, journal quality and research dynamics. CANCERS as the journal with the highest number of publications and THERANOSTICS as the journal with the highest impact factor among the journals with the highest number of publications in 10 indicates that these journals have influence and scholarly contributions in the field of NIR-PIT research. NAT MED as the journal with the highest citation frequency among the co-cited journals and the highest impact factor indicates a high academic reputation and influence in the field of NIR-PIT research, and the articles published in this journal are widely cited and recognized in the academic community. The dual map overlay of the journals provides a clear view of the development and evolution of the field of NIR-PIT research, helping researchers and journal editors to understand the dynamics of the field and the direction of development.

The co-cited reference analysis identifies references that have significant impact in a particular field or subject, and the most frequently cited publication in this study was a review article on newly developed photochemistry-based cancer therapy NIR-PIT published in 2019 (Mitsunaga et al., 2011), it provides direction for further research and suggests theoretical frameworks with far-reaching implications. Citation burst analysis assists in identifying documents that have attracted widespread attention over a given period of time, revealing emerging research themes or hotspots. Of the five publications bursting from 2021 to the present, two reported on clinical trials of RM-1929 PIT in rHNSCC (Tahara et al., 2021; Cognetti et al., 2021). Provides support for clinical trial data for NIR-PIT in the treatment of solid tumours with strategic importance for the direction of the research field. These data not only help the medical community and researchers to assess the actual effectiveness of the technology, also provide a scientific basis for further optimizing treatment protocols and guiding clinical practice.

### 4.2 Research hotspots and trends

In academic documents, keywords not only help in the retrieval of papers, also help to convey the core themes and content of the research. The keyword co-occurrence analysis can be used to identify the research hotspots, main research themes and the interrelation between the application of NIR-PIT in cancer therapy. To understand the correlation between different research themes and the focus of researchers by analyzing the frequently occurring keywords. In this study, we analyzed the keywords in the literature related to the field of NIR-PIT, in which the top 10 keywords in terms of frequency of occurrence, including near-infrared photoimmunotherapy (166), photodynamic therapy (61), monoclonal antibody (58), *in vivo* (50), cancer (46), expression (31), breast cancer (27), enhanced permeability (24), antibody (23), growth factor receptor (16). On the basis of keyword co-occurrence analysis, keyword clustering analysis and burst analysis were performed to further deeply excavate the research hotspots and research trends of the application of NIR-PIT in cancer treatment.

#### 4.2.1 NIR-PIT vs. PDT

NIR-PIT, as a derivation and optimization of PDT, has obvious advantages, including higher targeting and selectivity, enhanced immune response, reduced systemic side effects and broad applicability (Wakiyama et al., 2021). “Near-infrared photoimmunotherapy” (166), as the most frequently occurring keyword, is clearly a core research area within the research field theme. “Photodynamic therapy” (61) followed closely behind, indicating that PDT still has an important role in the application of NIR-PIT for the treatment of cancer.

NIR-PIT and PDT are both photoexcitation-based cancer treatments that rely on the excitation of photosensitizers to induce cell death, but there are significant differences in the way both work and the treatment efficacy. PDT is based on a photochemical reaction between light-activated molecules or photosensitizers, light and oxygen molecules. PDT involves two individually non-toxic components that combine to induce both cellular and tissue effects in an oxygen-dependent manner (Dolmans et al., 2003). The first component of PDT is the photosensitizer, a light-sensitive molecule localized to the target cells and/or tissues. The second component involves the application of specific wavelengths of light to activate the sensitizer. The photosensitizer transfers energy from light to molecular oxygen to produce reactive oxygen species (ROS) substances. The generated ROS cause oxidative damage to important biomolecules such as cell membranes, proteins and DNA, thus leading to the death of tumour cells (Bacellar et al., 2015). The photosensitizers used in PDT are a class of substances capable of generating ROS under light irradiation, and commonly used photosensitizers include porphyrin compounds, such as photosensitive violet, porphyrin derivatives, and nonporphyrin compounds (Diamond et al., 1972; Forbes et al., 1980). Indocyanine green (ICG) as one of the nonporphyrin photosensitizers used commonly in PDT. ICG relies primarily on the photothermal effect produced by excitation of NIR light, which leads to an increase in local tissue temperature, thereby triggering cell necrosis (Mahmut et al., 2023). In addition, ICG is also commonly used for *in vivo* imaging and diagnostics, with low photoexcitation efficiency and relatively insufficient targeting. PDT is irradiated with light of a specific wavelength, usually in the range of 600–800 nm (Kwiatkowski et al., 2018; Xiang et al., 2021). PDT causes oxidative damage and death of tumor cells by generating ROS and is usually applied to superficial tumors or tumor surfaces (Cramer et al., 2022). PDT is used as an effective cancer treatment, but there are some side effects and challenges. The most common complications include photosensitivity reactions, skin reactions, risk of infection, and allergic reactions (Aebisher et al., 2024). In the future, there is a need to improve the therapeutic efficacy of PDT and reduce side effects by improving photosensitizers, optimizing light sources, developing individualized treatment plans, and combining other therapeutic approaches.

NIR-PIT is based on three elements, mAb, photosensitizer, and NIR light to achieve accurate targeting of tumor cells, which produces physical effects and immune activation, leading to cell necrosis or immunogenic cell death (ICD) (Maruoka et al., 2020). The frequent occurrence of the keywords “monoclonal antibody” (58) and “antibody” (23) emphasizes the centrality of monoclonal antibody in NIR-PIT. mAb is a type of antibody that can specifically recognize and bind to a particular antigen (Zhou and Mascelli, 2011). In NIR-PIT, mAb is usually designed to target specific antigens on the surface of tumor cells to achieve highly specific recognition of tumors (Mahmut et al., 2023). Currently, research is focused on the development of APC that bind to different tumor markers, and mAb serves as an important carrier for targeted therapy in NIR-PIT. Therefore, antibody engineering and drug design technologies are the future direction of development, and the application of mAb will further promote the precision and efficacy of NIR-PIT, as well as expand the range of application.

Photosensitizers are usually a molecule that can be activated by irradiation with light of a specific wavelength, usually NIR light, and after coupling with a mAb, form a photosensitizer-antibody conjugates. When NIR light irradiates the photosensitizer-antibody conjugates on the surface of a cancer cell, the photosensitizer molecule absorbs light energy, triggering a series of physical effects that immediately induce massive cell death in the targeted tumor cell. IR700 dyes are commonly used as photosensitizers for NIR-PIT, causes direct cell membrane disruption under NIR light irradiation by conjugating tumor cell surface antigens through specific antibodies, inducing ICD, independent of ROS generation (Kobayashi and Choyke, 2019). This makes IR700 more suitable for use in hypoxic tumor microenvironments and enables the activation of anti-tumor immune responses with greater targeting and therapy specificity. The NIR light used in PIT, usually in the 700–900 nm wavelength range, has a good tissue penetration ability and can penetrate deeper into the tumor tissue (Xu et al., 2020). In NIR-PIT, these three elements cooperate with each other to form an effective therapy system. mAb ensures targeted delivery and accumulation of the photosensitizer, enhancing therapy specificity and selectivity. The photosensitizer is excited by NIR light which produces a physical effect that acts directly on the tumor cell membrane, ultimately leading to the destruction of the tumor cells. NIR light is the necessary light source to provide excitation of the photosensitizer and, due to its excellent tissue penetration, allows for the treatment of deep-seated tumors.

In general, PIT and PDT, as two different phototherapy, each has its own specific advantages and areas of application. NIR-PIT generates a synergistic effect of physical effect and immune activation, killing tumor cells by directly damaging the cell membrane through physical effect, while releasing antigens to further activate the systemic immune system. PIT combines the dual effects of local physical damage and systemic immune activation, and may have a more significant lasting killing effect on tumors. Therefore, NIR-PIT is suitable for a variety of solid tumors (Aung et al., 2018; Fukushima et al., 2022a; Matsuoka et al., 2022; Paraboschi et al., 2021), including tumors that are deeply located or difficult to be treated by traditional methods. In contrast, PDT is more suitable for the treatment of superficial tumors with relatively weak targeting and immune activation effects. PIT, despite the promising therapy it has demonstrated, still faces a number of side effects and challenges. Side effects focus on local and systemic reactions, including skin reactions, allergic reactions and immune system side effects. Current challenges include limitations in the depth of penetration of the light source, limitations in the targeting and selectivity of photosensitizers, and differences in individual therapy efficacy and therapy response. Further research and technological advances are needed to improve the treatment efficacy and safety of PIT. Therefore, future research will focus on optimizing photosensitizers, improving light source technology, optimizing the combined application of PIT with other therapeutic methods, forming comprehensive treatment protocols to achieve synergistic effects, and expanding the field of clinical applications of PIT in different diseases.

#### 4.2.2 NIR-PIT in cancer therapy

NIR-PIT, as an emerging approach to therapy, has shown potential application in the therapy of cancer. The frequency of the keyword “*in vivo*” (50) indicates the importance of the experimental model in the current NIR-PIT research, and *in vivo* experiments provide the basis for verifying the efficacy and safety of the new technology. The frequency of 24 occurrences of “enhanced permeability” indicates that enhanced drug delivery and tissue penetration are also the focus of current *in vivo* experiments. In addition, *in vivo* models serve an indispensable role in promoting the translation of NIR-PIT to the clinic, and researchers should pay attention to the design of rational animal experiments to verify the safety and efficacy of NIR-PIT, and more model-based studies in the future are expected to accelerate the clinical application of this technology.

The high frequency of the keywords “cancer” (46), “breast cancer” (27), “expression” (31), and “growth factor receptor” (16) reflect that NIR-PIT shows promising results in a wide range of cancer types, especially in breast cancer shows good promise. Different types of tumors express specific growth factor receptors, and by targeting these receptors, NIR-PIT can effectively kill tumor cells. With in-depth research on different cancer biomarkers and receptor expression, NIR-PIT is expected to play a role in more cancer types and achieve personalized treatment. The first human Phase 1/2 clinical trial of NIR-PIT using cetuximab-IR700 targeting EGFR in patients with inoperable recurrent head and neck tumours was successfully completed in 2017 (Mitsunaga et al., 2011). In 2020, the first antibody-photosensitizer conjugate, cetuximab saratolacan (previously known as RM-1929, commercial name: Akalux) has now been approved by the Japanese government for the treatment of unresectable locally advanced or recurrent head and neck cancer (Gomes-da-Silva et al., 2020). The use of NIR-PIT in head and neck tumors has become one of the most exemplary and intensively studied fields of this treatment technique. NIR-PIT is in testing as an international phase III clinical trial for locoregional rHNSCC patients (LUZERA-301, NCT03769506), with a fast track designation by the United States Food and Drug Administration (US-FDA) (Yamada et al., 2023), and expected to be approved in a few years. Nishikawa et al. (2022) reported the treatment of two patients with oropharyngeal lesions. Treatment responses were good in both cases with no severe adverse events and no functional disorders observed after HN-PIT. Makino et al. (2024) reported for the first time a case of, NIR-PIT for recurrent salivary duct carcinoma of the parotid gland, non-squamous cell carcinoma (non-SCC) of the head and neck region, and the results suggest that NIR-PIT may be effective in EGFR-positive non-SCC. EGFR expression was present in 71%–79% of salivary gland carcinomas (Locati et al., 2009) and 33.1% of salivary duct carcinomas (Takase et al. 2017), making NIR-PIT a viable strategy for the treatment of local recurrence in this population. The application of NIR-PIT in the treatment of head and neck tumors shows significant promise and distinct advantages, but the current study has a small sample size and limited long-term follow-up data, and larger studies are needed in the future to validate the long-term efficacy and safety of NIR-PIT.

Potential applications of NIR-PIT in other different cancer types. Since NIR light can activate photosensitizers that specifically aggregate in tumor cells without significantly damaging healthy brain tissues, thus realizing precise tumor ablation, this technology is particularly suitable for the treatment of refractory brain tumors such as malignant gliomas. Li et al. (2022a) proposed a novel nanotheranostic for a second near infrared (NIR-II) fluorescence imaging-guided combination PIT of GBM, which provides a new pathway for precision PIT of GBM. Nakamura et al. (2017) evaluated the therapeutic efficacy of NIR-PIT in a transgenic model of human EGFR-expressing spontaneous lung cancer (hEGFR-TL), and showed that the tumour volume ratio was significantly inhibited in the NIR-PIT group compared with control group (*p* < 0.01), and that NIR-PIT effectively treated a case of a spontaneous lung cancer in a hEGFR-TL transgenic mouse model. Yasui et al. (2021) developed a NIR-PIT technique targeting GPR87, showed that 54% of lung cancers and 100% of MPMs in surgical specimens exhibited high expression of GPR87. It showed therapeutic effects on lung cancer and MPM cell lines *in vitro* and in multiple models *in vivo*. NIR-PIT targeting GPR87 is a promising therapeutic approach for the treatment of thoracic cancer. Li et al. (2022b) constructed a hybrid cell model containing MRP1-positive H69AR cells and NIH/3T3 stromal cells, which showed that NIR-PIT successfully targeted and killed tumor cells. More importantly, NIR-PIT also led to a significant increase in tumor permeability, resulting in a 9-fold increase in the accumulation of liposomal doxorubicin (Doxil). Nakamura et al. (2017) developed a new phototherapy targeting Podoplanin (PDPN) for malignant pleural mesothelioma (MPM) using both NIR-PIT and anti-PDPN antibody, NZ-1. An APC consisting of NZ-1 and phthalocyanine dye was synthesized. The results showed that PDPN-targeted NIR-PIT produced significant anti-tumour effects in mouse model of MPM. Several animal model studies have demonstrated the significant inhibitory effect of NIR-PIT on thoracic tumors. Due to the deep location of the tumor and the many surrounding vital organs in thoracic tumors, the penetration depth of NIR light is limited and may be difficult to effectively reach the tumor site. Therefore, continuous development of photosensitizers with better tumor targeting and tissue penetration capabilities, as well as development of more advanced drug delivery systems, such as nanoparticles or targeted delivery systems, are needed in the future to improve the accumulation efficiency of photosensitizers in thoracic tumors. In addition, more large-scale clinical trials and long follow-up studies are needed to evaluate the long-term efficacy and safety of NIR-PIT in thoracic tumors.

The application of NIR-PIT in breast cancer has focused on the subtypes of breast cancer that express specific markers, such as HER2 positivity. Preliminary studies have shown that NIR-PIT is not only effective in reducing tumour volume, it also prevents recurrence and metastasis by inducing activation of the immune system. Yamaguchi et al. (2021) evaluated the efficacy of using the HER2 Affibody-IR700Dye conjugate on HER2 overexpressing breast cancer cells, including cells from brain metastases. Affibody is a small molecule protein with rapid clearance, high imaging contrast and good tumour penetration, as well as the ability to cross the blood-brain barrier, NIR-PIT using Affibodies has the potential to extend NIR-PIT to target cancers, including brain metastases. Further, investigated a combination of NIR-PIT for HER2-positive breast cancer cells (SK-BR3, MDA-MB361, and JIMT1) with HER2 Affibody-IR700Dye conjugate and trastuzumab-IR700Dye conjugate, result show that the combination of NIR-PIT with both HER2 Affibody and trastuzumab extends the targeting of NIR-PIT in HER2-positive breast cancer (Yamaguchi et al., 2021). Yamashita et al. (2023) explored the efficacy potential of NIR-PIT targeting HER2 using trastuzumab-IRDye700DX conjugate (Tra-IR700) in HER2-positive breast cancer. The results showed that NIR-PIT with Tra-IR700 induced highly selective therapy effect in a HER2-positive breast cancer model. NIR-PIT with Tra-IR700 holds promise as a novel treatment for HER2-positive cancers, including breast cancer and other HER2-positive cancers. (Fukushima et al., 2022b) investigated the efficacy of intercellular adhesion molecule-1 (ICAM-1) targeting NIR-PIT in the treatment of triple-negative breast cancer (TNBC). The study found that ICAM-1-targeted NIR-PIT significantly inhibited tumour growth and prolonged survival using a human TNBC xenograft mouse model. Therefore, ICAM-1-targeted NIR-PIT is a promising targeted therapy against TNBC and may be a good candidate for human trials. Although the outcome of NIR-PIT is optimistic in early animal trials, future large-scale and multi-centre clinical trials are needed to verify the efficacy and safety of NIR-PIT in breast cancer, and to provide a foundation for its wide application in the clinic. In addition, with the development of precision medicine, the application of NIR-PIT should be developed in the direction of individualized treatment, requiring tailor-made NIR-PIT treatment protocols based on patient-specific tumour types, molecular characteristics and treatment response, as well as combination therapy strategies, in order to improve the efficacy of the treatment and reduce the side effects.

## 5 Conclusion

In this study, based on bibliometric and visual analysis, a comprehensive analysis of the research hotspots and development trends of NIR-PIT in cancer treatment is presented. NIR-PIT, as an emerging cancer treatment, has received extensive attention in recent years in terms of molecularly targeted therapies and immune activation mechanisms, as well as combined therapy strategies in various solid tumors. The research hotspots focus on the development of antibody-photoabsorber conjugate, optimization of light dosage parameters and enhancement of therapeutic targeting. Trend analysis suggests that future research may further focus on improving the efficacy and safety of NIR-PIT in cancer therapy, as well as developing novel photosensitizers and combination therapy regimens and exploring its potential application in a variety of solid tumors, which provides an important reference and guidance for the application of NIR-PIT in clinical translation.

## Data Availability

The original contributions presented in the study are included in the article/Supplementary Material, further inquiries can be directed to the corresponding author.
