# Supplementary material for: Near-infrared photoimmunotherapy in cancer treatment: a bibliometric and visual analysis
Source: Front Pharmacol. 2024 Oct 21;15:1485242. doi: 10.3389/fphar.2024.1485242 (PMC11533137; doi:10.3389/fphar.2024.1485242)
Supplement: Supplementary file 1 [file DataSheet2.PDF]

Table S1. Annual and cumulative number of relevant publications

Rank	Year	Number of publications	Cumulative total
1	2011	1	1
2	2012	2	3
3	2013	1	4
4	2014	3	7
5	2015	7	14
6	2016	21	35
7	2017	21	56
8	2018	21	77
9	2019	24	101
10	2020	30	131
11	2021	40	171
12	2022	33	204
13	2023	26	230
14	2024	15	245

Table S2. Top 10 institution for relevant publications

Rank	Institutions	Count	Centrality
1	National Institutes of Health (NIH) - USA	114	0.11
2	Nagoya University	15	0.01
3	Hokkaido University	12	0.01
4	Japan Science & Technology Agency (JST)	10	0
5	Harvard University	7	0.02
6	Okayama University	7	0.01
7	Harvard Medical School	6	0.01
8	Frederick National Laboratory for Cancer Research	6	0
9	Massachusetts General Hospital	6	0.01
10	Niigata University	5	0.03

TableS3. Top 10 keywords for related publications

Rank	Keywords	Count	Year	Centrality
1	near-infrared photoimmunotherapy	166	2013	0
2	photodynamic therapy	61	2011	0.02
3	monoclonal antibody	58	2011	0.11
4	in vivo	50	2013	0.25
5	cancer	46	2015	0.09
6	expression	31	2015	0.08
7	breast cancer	27	2014	0.2
8	enhanced permeability	24	2015	0.05
9	antibody	23	2016	0.19
