# Supplementary material for: Near-infrared photoimmunotherapy in cancer treatment: a bibliometric and visual analysis
Source: Front Pharmacol. 2024 Oct 21;15:1485242. doi: 10.3389/fphar.2024.1485242 (PMC11533137; doi:10.3389/fphar.2024.1485242)
Supplement: Supplementary file 2 [file DataSheet1.PDF]

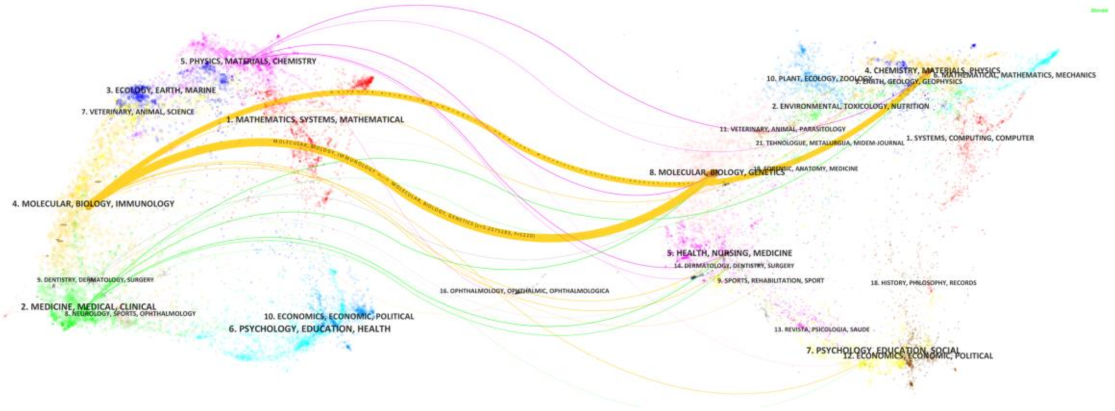

Figure S1. The dual-map overlay of journals.

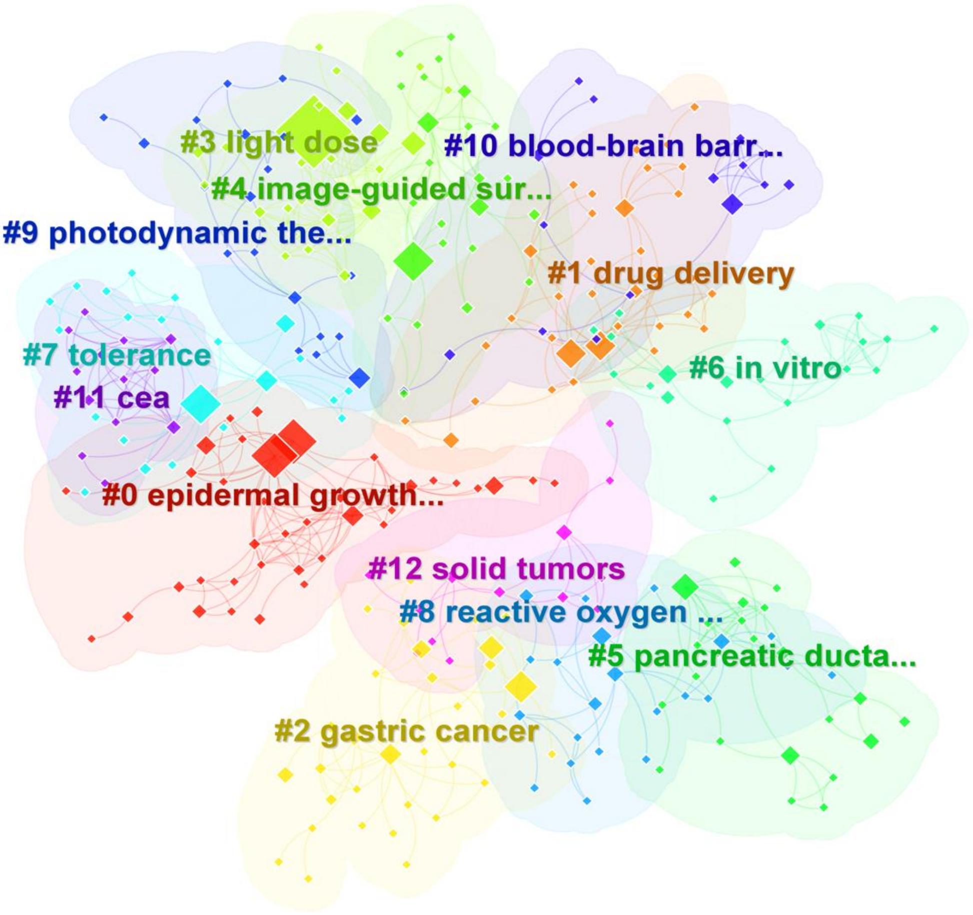

Figure S2. Visual mapping of keyword cluster.

Top 24 Keywords with the Strongest Citation Bursts

Keywords	Year	Strength	Begin	End
ovarian cancer	2011	3.19	**2011**	2016
apoptosis	2012	2.37	**2012**	2017
mouse	2012	2.05	**2012**	2016
gfp	2012	1.94	**2012**	2014
cancer cells	2013	3.18	**2013**	2017
efficacy	2013	3.13	**2013**	2015
growth	2013	2.03	**2013**	2015
near infrared light	2014	2.16	**2014**	2016
fluorescence guided surgery	2015	2.25	**2015**	2016
pancreatic cancer	2015	1.97	**2015**	2018
enhanced permeability	2015	3.63	**2016**	2017
molecular imaging	2016	2.32	**2016**	2019
drug delivery	2016	1.98	**2016**	2020
gastric cancer	2018	2.13	**2018**	2019
mouse model	2018	2.01	**2018**	2019
surgery	2018	2.01	**2018**	2019
nanoparticles	2018	1.95	**2018**	2021
phase ii trial	2019	1.93	**2019**	2020
monoclonal antibodies	2020	2.19	**2020**	2021
cytotoxicity	2020	2.14	**2020**	2022
epidermal growth factor	2021	2.09	**2021**	2022
membrane	2022	3.71	**2022**	2024
cancer	2015	2.81	**2022**	2024
therapy	2013	1.93	**2022**	2024

Figure S3. Top 24 keywords with the strongest citation bursts.
